# **Knowledge Mobilization and Academic Health Science Centres in Australia** Comment on "Academic Health Science Centres as Vehicles for Knowledge Mobilisation in Australia? A Qualitative Study"

**DOI:** 10.34172/ijhpm.2021.125

**Published:** 2021-09-06

**Authors:** Dimitrios Spyridonidis

**Affiliations:** Warwick Business School, Coventry, UK.

**Keywords:** Knowledge Mobilization, Leadership, Impact, Australia, Human Resource Management

## Abstract

Extant research on knowledge mobilization points to barriers and opportunities for innovation. Edelman et al paper "Academic Health Science Centres as Vehicles for Knowledge Mobilisation in Australia? A Qualitative Study" builds nicely on the existing knowledge base by evaluating the early stages of organisational development of Academic Health Science Centers in Australia. This commentary discusses their research findings by drawing on relevant themes including knowledge mobilization initiatives that have been developed globally to bridge the research-practice gap and knowledge brokering roles for service improvement. Following which, the commentary draws on organizational capabilities literature for knowledge brokering to happen, the latter including the need for measuring implementation fidelity amongst other capabilities. Finally, building on Edelman et al call for more attention to action-oriented roles and knowledge mobilization processes to deliver strategic goals the commentary concludes with a note for collective leadership as an enabler of knowledge mobilization with impact and at scale.

 Healthcare represents a setting in which research-based clinical knowledge is expected to inform frontline clinical practice to enhance patient outcomes.^1^ Following which there has followed a plethora of knowledge mobilization initiatives globally. Australia has followed suit, seeking to bring together universities and healthcare organisations through Academic Health Science Networks, and Academic Health Science Centres (AHSCs) as Vehicles for Knowledge Mobilisation in Australia.^2^

 Whilst strong on their promise to translate research-based knowledge into practice, early indications suggest the progress of knowledge mobilization initiatives in healthcare have not been smooth, with evidence of conflict, as well as collaboration, across academic, managerial and clinical practice communities regarding how research-based knowledge is best mobilised. More critically informed evaluations emanating from business and management researchers about Collaborations for Leadership in Applied Health Research and Care,^3-6^ in the English National Health Service (NHS), highlight contestation about types of knowledge, methods for generating such knowledge, and the roles enacted for knowledge mobilisation amongst the many actors involved in translational health research initiatives.

 Edelman et al^2^ investigation points to the same direction. AHSCs as vehicles for knowledge mobilisation in Australia reports on a qualitative study that explores whether and how they deliver impact by examining and comparing the early development of four Australian AHSCs to explore how they are enacting their impact-focused role. The research founds that while AHSCs in Australia are in an emergent state of development they are following different pathways while there is a dominant focus on structure and governance, as opposed to action-oriented roles and processes to deliver strategic goals of high-quality research, education and care.

 Internationally, there is no surprise that knowledge mobilization initiatives have developed to bridge the research-practice gap through ‘push,’ ‘pull,’ and ‘exchange’ strategies.^7^ ‘Push’ strategies posit that knowledge producers offer rigorous research, which then needs to ‘push’ outwards to knowledge users. ‘Push’ strategies focus on creating, sharing, or spreading, both, actively and proactively, research out to prospective users. In contrast, the emphasis on ‘pull’ strategies is on increasing demand for the acceptance of new knowledge by concentrating on the needs of prospective users. Finally, ‘exchange’ strategies, which require engagement of multiple stakeholders in both research and implementation processes, have been proposed as an alternative to the ‘pull’ and ‘push’ strategies.

 Edelman et al suggest building research capacity and literacy among local clinicians and community was also described in one AHSC as a strategy to make research more responsive to community priorities. These priorities call attention to the importance of organizational systems and structures, such as financial and human resource management, organizational structures, organizational and systems governance, organizational cultures, and professional power, in shaping impact of academic research upon practice.^6^ Edelman et al suggest shifting from researcher-led models to research co-produced. One panacea presented to enhance the effectiveness of research co-produced with academic-practitioner collaborations is the development of knowledge brokering roles to engage practitioners.^8^ The practitioner’s role as knowledge brokers should be allowed at all stages of the decision-making process so that the knowledge they hold is transformed and exploited. Their role as knowledge brokers in formal translational health research structures facilitates this. However, studies highlight knowledge brokers merely generate a ‘ripple in the pond’ in a healthcare system consisting of tens of thousands of professionals, and a myriad of health and social care organizations.^9^ Even enthusiastic proponents of knowledge brokering, such as Rowley et al,^9^ highlight that the divide between Universities and the NHS can stymie translation of research into practice. Following which, in healthcare systems, they argue there needs to be individual, group and organizational interest in translating research evidence, as well as incentives to participate for all concerned. To justify the time and commitment of those involved, there also need to be tangible outcomes that are valued alike by researchers and practitioners. There also needs to be incentives and opportunities for clinicians to participate in translational research. Research capacity building should take into account how human resource interventions, namely, performance management, job design, and training and development shape enactment of knowledge brokering roles by professionals.^10^ Resent research points to the role of human resource management practices that shape the dynamics of knowledge brokering for service improvement, in particular practitioners’ perceptions of the effect of human resource practices upon their legitimacy and identity shape their attitudes and behavior toward knowledge brokering.^10^

 Edelman et al found that the language of knowledge mobilisation was not generally used by interviewees. Most used the narrower concept of translation, referring predominantly to researcher-produced knowledge and its application in clinical contexts. It is necessary to remember that knowledge mobilization is not a rational linear process, nor is it a single decision or event, while acquiring knowledge is not the same as adopting it.^11^ Instead, critics argue, knowledge mobilization is more dynamic and reciprocal, and they emphasise a process of social interaction, meaning negotiation and exchange between research producers and users.^6^

 Edelman et al found that establishing high-level governance structures concerned with ensuring adequate representation of participating partners to form a basis for effective collaboration was important. Those leading knowledge mobilization initiatives need to fully reflect what is working and why, for whom, what needs to be adapted as we scale up, and how we might glean resource for any potential scale up.^12^ Balancing collaboration and competition between partners present a challenge, as does identifying appropriate ways to evaluate impact. Regarding innovation diffusion, evaluation of impact is most relevant, underpinned by organizational capabilities as they related to formal knowledge exchange mechanisms, such as written policies, procedures and manuals designed to facilitate transfer of codified knowledge, but also to environmental incentives that shape priorities. Evaluation of impact can be systematized by the formalization and routinization of organizational routines^12^ including the social relationships between the organization and its external partners.^12^ What this means in essence is that an organization to develop impact it requires capabilities to recognise the value of new information, assimilate and apply it at scale. Overall, an organization with the ability to deliver impact, it is suggested, can handle knowledge stocks and flows better^12^ is more pro-active in securing and embedding new and useable knowledge and produce a dynamic organizational capability, that is the ability to acquire and assimilate knowledge and the ability to put newly acquired knowledge into action within the organization through transformation (the development and piloting of an intervention) and exploitation (scaling up of that intervention). Effective knowledge mobilization programmes require the implementation of research into practice with fidelity, defined as the degree to which a programme is implemented as planned. It represents the quality and integrity of the interventions as conceived by the developers.^13^ Programme fidelity contributes to an understanding of why some interventions can succeed in one context and fail in others). Implementation research posits that fidelity needs to be measured and documented carefully and reliably in order to increase confidence in implementation progress.^13^ Business intelligence systems are important too but there is a need to go beyond information systems solutions, since for knowledge mobilisation data analysts within the organisation need to collaborate with managers and frontline clinicians to ensure service improvement is derived from robust data held within healthcare organisations and systems.^12^

 Implementation research, however, has suggested several factors that may undermine strategies for measuring implementation fidelity. For example, clinicians may resist the use of standardized tools and outcome measures because they perceive measurement to be an unrealistic burden on their practice and that it is too far removed from their clinical realities.^14^ To this end it is welcoming to note that Edelman et al are making a call for action-oriented roles and processes to deliver strategic goals of knowledge mobilization, which often are ambiguous and contested processes^6^with interactions needing to allow for an understanding of how individuals and groups negotiate ambiguity, reach consensus and make sense of new knowledge. Knowledge mobilization can be identified as enacted across two broadly defined phases (local implementation and scale up), with leadership in the second phase moving away from a purely individualistic or heroic action towards enactment by diverse actors.^15^

## Ethical issues

 Not applicable.

## Competing interests

 Author declares that he has no competing interests.

## Author’s contribution

 DS is the single author of the paper.
